# A case in which the tip of a plastic stent placed to prevent recurrence of walled‐off necrosis penetrated the bile duct and formed a stent–stone complex

**DOI:** 10.1002/deo2.220

**Published:** 2023-03-28

**Authors:** Fumi Sakuma, Atsushi Irisawa, Satoaki Noguchi, Yoko Abe, Koki Hoshi, Akira Yamamiya, Kazunori Nagashima, Ken Kashima, Yasuhito Kunogi, Koh Fukushi, Kohei Inaba, Ken Oike, Saori Furuki, Keiichi Tominaga, Kenichi Goda

**Affiliations:** ^1^ Department of Gastroenterology Dokkyo Medical University Tochigi Japan

**Keywords:** adverse events, bile duct, fistula, stent stone complex, walled‐off necrosis

## Abstract

A woman in her 60s underwent endoscopic sphincterotomy for choledocholithiasis. Unfortunately, post‐endoscopic retrograde cholangiopancreatography pancreatitis occurred. In addition, huge walled‐off necrosis (WON) appeared as a late complication. For the infected WON, endoscopic ultrasound‐guided fistuloplasty and endoscopic necrosectomy were performed, and a double pigtail plastic stent (PS) (7Fr, 7 cm) was placed to prevent a recurrence. Plain computed tomography conducted two years later showed that the stent implanted for WON had deviated. The distal end of the stent was found to have migrated into the bile duct. In addition, common bile duct stones with stents as nuclei were observed. Upon performing endoscopic retrograde cholangiography, it was revealed that the stent tip perforated the distal bile duct just above the papilla. After removal of the stent using grasping forceps, we made an incision between the duodenal – bile duct fistula and bile duct orifice using a sphincterotome. Then, the stone was removed by a balloon catheter. Although such late adverse events are rare occurrences, placement of long‐term PS after treatment of WON should be followed up regularly with imaging examination, and if there is no recurrence for several months, removal of the PS at that point may be considered.

## INTRODUCTION

Walled‐off‐necrosis (WON) after necrotizing acute pancreatitis is often difficult to control using conservative treatment alone. Endoscopic drainage with a step‐up approach is the standard treatment. To prevent a recurrence, a plastic stent (PS) is often placed after the reduction of WON, with few reports of adverse events due to long‐term placement of PS. This report described a case of duodenal biliary fistula formation with a stent–stone complex in the bile duct caused by the dislocation of a double pigtail PS placed after an endoscopic necrosectomy of WON.

## CASE REPORT

**FIGURE 1:**
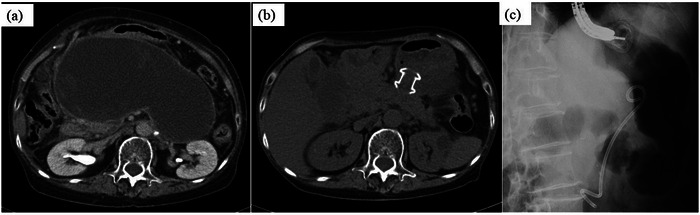
Contrast‐enhanced computed tomography. A huge walled‐off necrosis is in the abdominal cavity. (a) Abdominal plain computer tomography after lumen‐apposing metal stent implantation and treatment showing reduction of walled‐off necrosis. (b) The lumen‐apposing metal stent was removed. A double pigtail plastic stent (7Fr, 7 cm) was implanted.

A woman in her 60s underwent endoscopic sphincterotomy for choledocholithiasis. Unfortunately, post‐endoscopic retrograde cholangiopancreatography (ERCP) pancreatitis occurred. In addition, WON appeared as a late complication (Figure 1a). The patient complained of appetite loss, fever, and leg edema due to inferior vena cava compression by the huge WON, therefore, a lumen‐apposing metal stent was placed for the WON. Endoscopic ultrasound‐guided fistuloplasty between the stomach and the WON was performed. However, it was difficult to control the WON, hence, endoscopic necrosectomy was performed additionally based on a step‐up approach.

**FIGURE 2:**
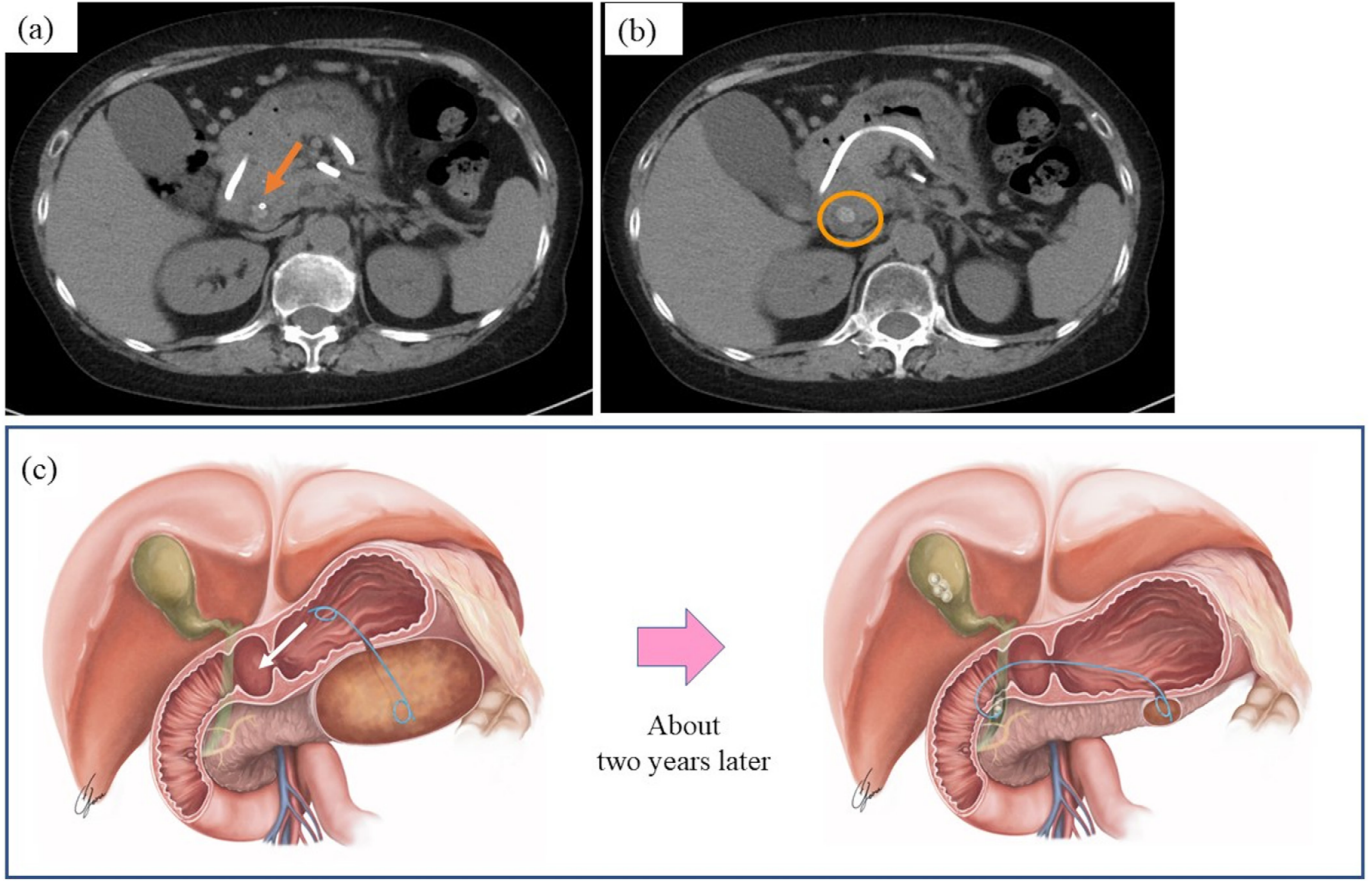
Abdominal plain computed tomography (CT) and sherma. (a) The stent tip is visible in the common bile duct (arrow). (b) Common bile duct stones with stents as nuclei were also observed (circled part). (c) Schema that the stent tip penetrated the bile duct and formed a stent–stone complex. The stomach end of the pigtail stent entered the duodenum and penetrated the bile duct due to the reduced walled‐off necrosis. The tip of the stent in the walled‐off necrosis was still barely inside the walled‐off necrosis.

The lumen‐apposing metal stent was removed 2 months after implantation because the WON had become smaller (Figure 1b). Subsequently, a double pigtail PS (7Fr, 7 cm) was placed to prevent recurrence (Figure 1c). After discharge, she was consulted every three months and had imaging examinations every 6–8 months. The patient was followed up without recurrence of WON, but about two years after the end of WON treatment, plain abdominal computed tomography showed that the PS placed in the WON had deviated. The distal end of the PS was found to have migrated into the bile duct via the duodenal wall (Figure 2a). Concurrently, a choledocholithiasis with the PS as nuclei was also observed (Figure 2b,c). However, she was asymptomatic. Blood tests showed no elevation of hepatobiliary enzymes or inflammatory markers.

**FIGURE 3:**
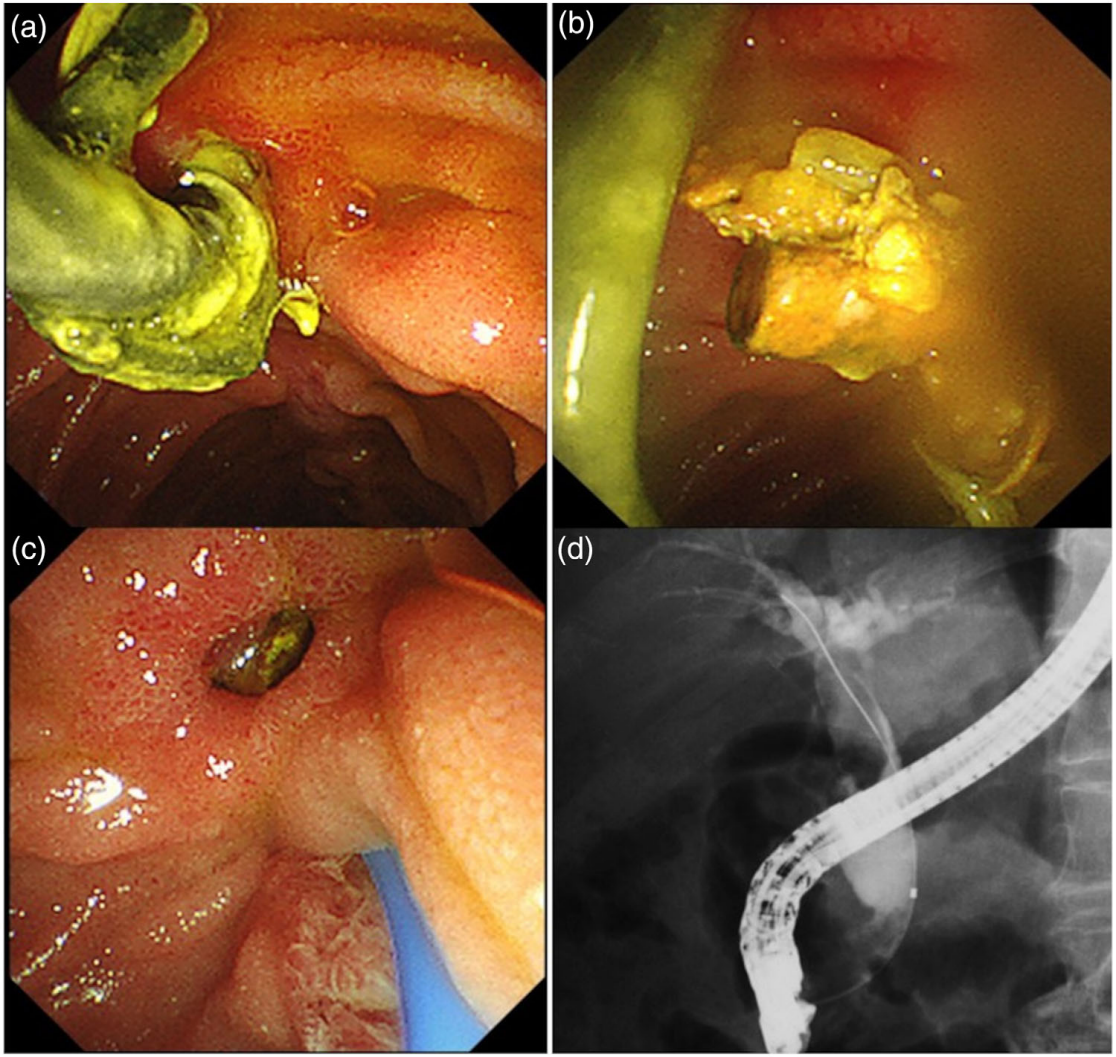
Endoscopic retrograde cholangiography. (a) The stent tip perforated the distal bile duct just above the major papilla. (b) The tip of the stent removed via the fistula. (c) The duodenal–bile duct fistula. (d) Cholangiography through the vater papilla revealed a translucent image caused by a common bile duct stone.

**FIGURE 4:**
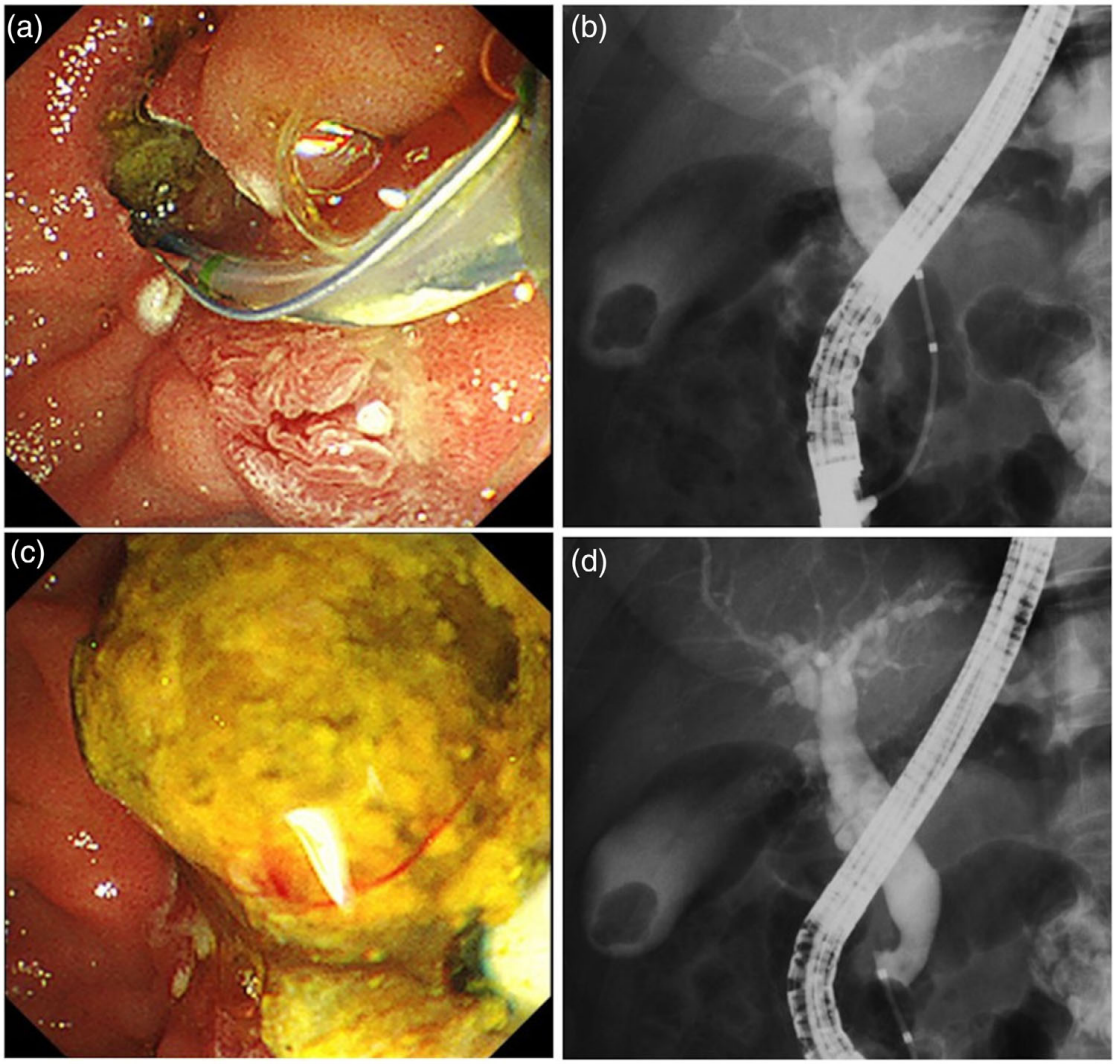
Endoscopic retrograde cholangiography. (a) After endoscopic sphincterotomy was done, the duodenal–bile duct fistula and bile duct orifice were connected. (b) Balloon catheter was used to remove the stone. (c) It was confirmed using cholangiography that no stone persisted.

ERCP was performed (Videos S1 and S2) on the fifth day of admission. The stent tip penetrated the distal bile duct just above the major papilla (Figure 3a). The PS was removed gently with grasping forceps, and then the duodenal‐bile duct fistula was visible (Figure 3b,c). Cholangiography through the vater papilla showed a translucent image attributable to choledocholithiasis (Figure 3d). We made an incision between the duodenal bile duct fistula and the bile duct orifice using a sphincterotome (Figure 4a). The stone was removed using a balloon catheter (Figures 4b,c). The procedure was completed after confirmation by cholangiography that no stone remained (Figure 4d). The patient was discharged on the 16th day after admission and has had no recurrence of WON or common bile duct stones for 1 year.

## DISCUSSION

WON‐derived acute necrotizing pancreatitis is a severe late adverse event. Endoscopic therapy with a step‐up approach is recommended because of its lower incidence of severe complications and lower short‐term mortality than those using surgical drainage.^1^, ^2^ An earlier reported randomized controlled trial^3^ showed that the relapse rate of pancreatic‐fluid collection including WON was higher in patients whose stents were removed after pancreatic‐fluid collection resolution than in patients whose stents were left in place. As stent removal was a risk factor, it was recommended that the stent be left in place for as long as possible. In the report, no patient had any severe adverse event, although the median observation period was short at 14 months. Although it is considered so many cases in which patients can be followed up with long‐term PS placement after the disappearance of WON, a few cases of gastrointestinal perforation have been reported.^4^, ^5^ On the other hand, there are no reports of bile duct duodenal fistula formation as in the present case. Considering the possibility that such a late adverse event may develop over a long period of time, it will be necessary to follow up.

A biliary stent can act as a nidus for new biliary stone formation around the PS after long‐term placement, called an stent–stone complex, first reported in 2007.^6^ One of the risk factors for stent–stone complex is known to be a long‐term stenting period of more than 300 days.^7^ Several cases have been reported in which large stones formed because of long‐term stenting of the bile duct. Although ERCP was attempted to remove the stone, surgical treatment may be required.^8^, ^9^ Fortunately, we could remove the stent and stone by ERCP‐related procedure. In our case, it is unclear when the PS penetrated the common bile duct. However, plain abdominal computed tomography imaging at about 8 months before showed that the PS was almost dislocated with the reduction of WON. In this computed tomography, there was no finding of the PS tip migrating into the bile duct via the duodenal wall or choledocholithiasis. Therefore, it was thought to have occurred after that time. From our case, it was considered that regular imaging after WON treatment with PS indwelling is very important not only for the prevention of recurrence of WON but also for monitoring the absence of complications due to long‐term placement of PS.

Our case was very rare and fortunately, this patient was treated endoscopically. However, if the stent tip had penetrated the peritoneal/retroperitoneal space through the duodenal wall, surgical treatment may have been required. We also placed the 7 cm double pigtail PS to prevent the recurrence of WON, but the 4 cm PS might have been used in anticipation of WON reduction. In cases with long‐term PS placement after treatment of WON, routine follow‐up with imaging examination should be performed. If there is no recurrence for several months, removal of the PS at that point may be considered.

## CONFLICT OF INTEREST STATEMENT

None.

## ETHICS STATEMENT

This case report was conducted in accordance with the ethical standards established in the 1964 Declaration of Helsinki and its later amendments.

## Supporting information

Video S1

Video S2
